# Biomarkers of Oxidative and Radical Stress

**DOI:** 10.3390/biom14020194

**Published:** 2024-02-05

**Authors:** Chryssostomos Chatgilialoglu

**Affiliations:** 1 Institute for Organic Synthesis and Photoreactivity, National Research Council (CNR), 40129 Bologna, Italy; chrys@isof.cnr.it; 2 Center for Advanced Technologies, Adam Mickiewicz University, 61–614 Poznań, Poland

## 1. Reactive Species, Oxidative Damage, and Biomarkers 

**Figure 1:**
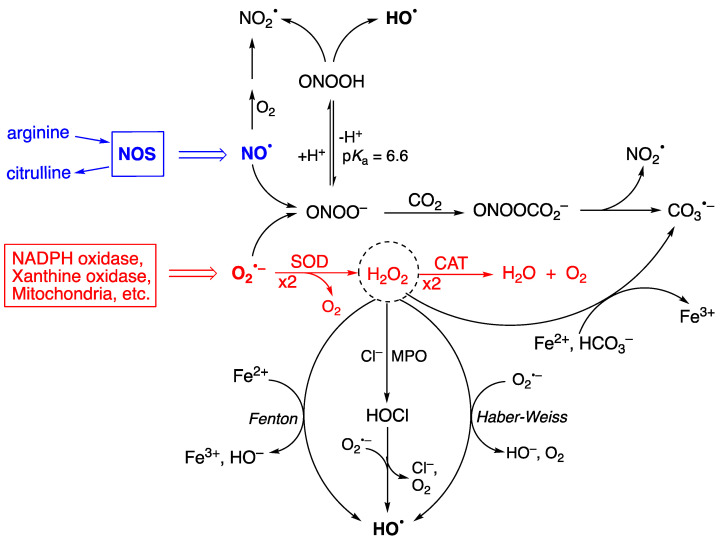
The enzymes SOD and CAT control the production of superoxide radical anion (**O_2_^•−^**) (see red color). The processes that generate HO^•^ are the Fenton and Haber–Weiss reactions of H_2_O_2_, the reduction of HOCl by O_2_^•−^, and the spontaneous decomposition of ONOOH, whereas the processes that generate CO_3_^•−^ are the decomposition of ONOOH and H_2_O_2_ reacting with Fe^2+^ and bicarbonate.

Reactive oxygen and nitrogen species (ROS/RNS) are generated as a result of normal intracellular metabolism. Their physiological roles in biology related to redox signaling mean that they participate in the modulation of apoptosis, stress responses, and proliferation [1,2,3]. ROS/RNS species include small molecules such as hydrogen peroxide (H_2_O_2_), peroxynitrite (ONOO^−^), and hypohalous acids (HOCl or HOBr), as well as radicals such as superoxide radical anion (O_2_^•−^), nitric oxide (NO^•^), hydroxyl radical (HO^•^), nitrogen dioxide radical (NO_2_^•^), and carbonate radical anion (CO_3_^•−^) [4,5]. The two progenitors of the ROS network are O_2_^•−^ and NO^•^ (Figure 1). Their concentrations are ~0.1 nM O_2_^•−^ and ~10 nM NO^•^ under physiological conditions, but these concentrations can increase by up to 100-fold during inflammatory response, having a negative effect by causing damage to biomolecules [6]. The enzymes superoxide dismutase (SOD) and catalase (CAT) control the production of O_2_^•−^, first by dismutation to H_2_O_2_ and O_2_ and, secondly, by transforming H_2_O_2_ to water and oxygen (see Figure 1—red color) [7]. Figure 1 also shows the main pathways via which other biologically important free radicals can be produced, either via H_2_O_2_ or as a consequence of peroxynitrite ONOO^−^ formation [8]. H_2_O_2_ is at the crossroads of several pathways for the formation of HO^•^, with the main ones being Fenton reaction (Fe^2+^ and H_2_O_2_) [9], Haber–Weiss reaction (O_2_^•−^ and H_2_O_2_), and the reduction of previously formed HOCl or HOBr by superoxide radicals. The spontaneous decomposition of ONOOH also produces HO^•^. ONOO^−^ reacts with CO_2_, and the resulting adduct rapidly decomposes to NO_2_^•^ and CO_3_^•−^. The regulation of ROS and related species, as well as oxidative repair, becomes less efficient with aging, resulting in the accumulation of ROS-derived damage. The *free radical theory of aging* claims that ROS-derived damage contributes to the functional decline of organ systems and predisposes those affected to pathologies such as cancer, as well as cardiovascular and neurodegenerative diseases [10]. 

**Figure 2:**
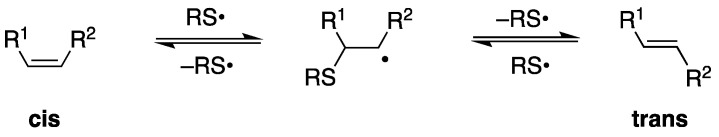
Thiyl radical-catalyzed cis–trans isomerization of a monounsaturated fatty acid moiety.

It is also worth mentioning that small reactive sulfur species (RSS) like H_2_S and its congeners or H_2_S-derived radicals are emerging players in biological processes, particularly those related to mitochondria chemistry [11]. In chemical biology, it is believed that molecule modification/damage is due mainly to the activation of ROS through RSS generation. Regarding the reactivity of free radicals and, in particular, thiyl radicals (RS^•^) towards biomolecules, there is the well-known cis–trans isomerization of double bonds in unsaturated fatty acids moieties, which leads to the alteration of these biomolecules without resulting in oxidative stress products [12]. Figure 2 shows the reaction mechanism for this transformation, which consists of the reversible addition of RS^•^ to the cis double bond, forming the trans geometry as the thermodynamically favorable structure. It is worth noting that the radical RS^•^ acts as a catalyst for cis–trans isomerization, and the isomerization mechanism occurs on polyunsaturated substrates as a step-by-step process (i.e., each isolated double bond behaves independently). 

**Figure 3:**
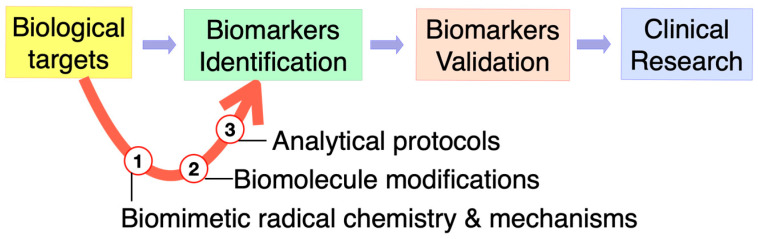
Omics technologies and the role of biomimetic radical chemistry in biomarker discovery.

Biomarkers and their products are substances, structures, or processes that can be measured in the body. They can be used to influence or predict the incidence of an outcome or a disease. Some of the reactive species mentioned above can damage organs, tissues, and cells by oxidizing DNA, proteins, and lipids, thereby resulting in diseases. The enormous importance of oxidative and free radical chemistry for a variety of biological processes, including aging and inflammation, has motivated researchers to try to understand the related mechanistic steps at the molecular level with the development of related biomarkers. The identification of modified biomolecules has a diagnostic value for the evaluation of in vivo damage. Therefore, the development of biomarkers through biomolecule modification and characterization by analytical protocols, followed by biomarker validation and extension to clinical research, have important applications in medicine and therapeutical approaches (Figure 3).

This Special Issue covers various aspects of biomarker research, from biomarker identification, including chemical reactivity and analytical procedures, to biomarker validation and pre-clinical applications. Examples include DNA oxidation products, peptide and protein modifications, lipid peroxidation and isomerization, and defense and repair strategies. 

## 2. Brief Overview of the Special Issue

### 2.1. Biomarkers of DNA Damage

**Figure 4:**
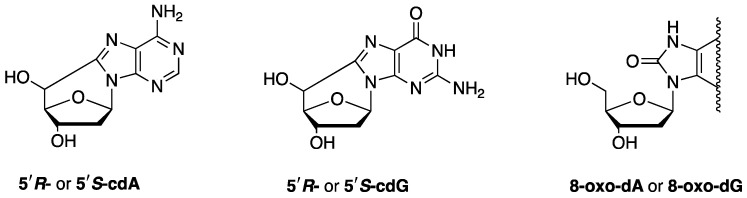
The structures of 5′,8-cyclo-2′-deoxyadenosine (cdA), 5′,8-cyclo-2′-deoxyguanosine (cdG), and 8-oxo-7,8-dihydro-2′-deoxypurine (8-oxo-dG or 8-oxo-dG).

Purine 5′,8-cyclo-2′-deoxynucleosides (cPu) are solely generated by the attack of HO^•^ radicals on purine moieties via C5′-radical chemistry, resulting in the formation of an additional C5′−C8 covalent bond; 5′,8-cyclo-2′-deoxyadenosine (cdA) and 5′,8-cyclo-2′-deoxyguanosine (cdG) exist in the 5′*R* and 5′*S* configurations (Figure 4). cPu can be removed only by the nucleotide excision repair (NER) pathway, and different repair efficiencies of *R* and *S* diastereoisomers have been detected [8]. On the contrary, the well-known 8-oxo-purine (8-oxo-Pu) lesions, namely, 8-oxo-7,8-dihydro-2′-deoxyadenosine (8-oxo-dA) and 8-oxo-7,8-dihydro-2′-deoxyguanosine (8-oxo-dG), derive from the oxidation at the C8 position of adenine and guanine caused by a variety of reactive oxygen species (ROS) and can be repaired by base excision repair (BER) [8]. 

Two articles in this Special Issue deal with the simultaneous measurement of six purine lesions in DNA samples using LC-MS/MS protocol. The first of these articles reports on Cockayne syndrome (CS) cell lines [13]. In particular, the six purine lesions were ascertained in the mtDNA of wild-type CSA and CSB cells and defective counterparts in comparison with the corresponding total nDNA. The 8–oxo–Pu levels were found to be in the range of 25–50 lesions/10^7^ nucleotides in both mtDNA and nDNA. The four cPu were undetectable in mtDNA both in defective cells and in the wt counterparts (CSA and CSB), contrary to their detection in nDNA, indicating the absence of HO^•^ reactivity within mtDNA. Additional tailored in vitro experiments demonstrated a higher resistance to HO^•^ attack for mtDNA in comparison with nDNA associated with their different DNA helical topologies. The second of these articles reports on the brain tissue of mice [14]. A murine model of immunodeficient (SCID) xenografted young (4 weeks old) and old (17 weeks old) mice was compared with corresponding controls without tumor implantation. Both cPu and 8-oxo-cPu formation was evaluated in this study to compare the effect of tumor development and gather information on the aging process. Progressive DNA damage due to age and tumoral conditions was confirmed by raised levels of 5′*S*-cdG and 5′*S*-cdA.

### 2.2. Biomarkers of Lipid Damage

The above-mentioned brain tissue of young (4 weeks old) and old (17 weeks old) mice, in parallel to DNA damage, was also evaluated to ascertain changes in lipid content [14]. The remodeling of fatty acids involved a diminution of palmitic acid accompanied by an increase in arachidonic acid, along both age and tumor progressions, causing increases in the unsaturation index, the peroxidation index, and total TFA, indicators of increased oxidative and free radical reactivity. The authors concluded that under aging and disease progressions, membranes are not spectators, and targeted strategies are needed for preserving their molecular integrity [14].

Lipid peroxidation (LP), an important type of oxidative/radical damage in biological systems, is associated with a large number of pathological conditions, from atherosclerosis and cardiovascular diseases to neurological disorders and cancer [15]. Moreover, ferroptosis is a caspase-independent type of cell death triggered by iron-dependent LP [16]. The chemistry of LP, with radical chain-propagation reactions, is reviewed in [17] in this Special Issue, with a focus on the kinetics of various processes which help us to understand the mechanisms and efficacy of antioxidant strategies. The author of this article also describes the LP products that are commonly used as biomarkers to monitor the damage of biological systems, as well as their ability as electrophiles to alter other biomolecules such as proteins. An overview of the biological consequences of LP is presented, with an emphasis on membrane integrity and function, cell signaling, cancer, neurological disorders, and ferroptosis [17].

4-hydroxynonenal (4-HNE) is the quintessential biomarker for the oxidative degradation of polyunsaturated fatty acids (PUFA). The relationship between 4-HNE and the metabolomic profiling of patients with prostate cancer is reported on in [18]. In this study, measurements of 4-HNE–protein adducts in prostate cancer tissues and plasma samples from prostate cancer patients were obtained and compared with samples collected from healthy controls. The results indicated an absence of 4-HNE–protein adducts in prostate carcinoma tissues but increased 4-HNE–protein levels in the plasma of these patients, associated with different long-chain and medium-chain fatty acids with the presence of prostate cancer. The metabolic pathway of unsaturated fatty acid biosynthesis was found to be significantly affected by 4-HNE 18].

Plasmalogens are an important class of membrane phospholipids; in red blood cells (RBCs), their content reaches 15–20% and increases in tissues like the heart (32–50%), brain (20–50%), and spermatozoa (55%). Plasmalogens are characterized by having the following two fatty acids: one unsaturated fatty acid linked to the glycerol moiety through a cis-vinyl ether function and the other PUFA residue linked through an acyl function. An article in this Special Issue reports on the radical reactivity and identification of TFA [19]. In addition to the reported optimal transesterification procedure, it was demonstrated that the vinyl ether reactivity is similar to the reactivity of the arachidonic moiety that contains four C–C double bonds in cis–trans isomerization by thiyl radicals. Furthermore, a biomimetic Fenton-like model involving plasmalogen-containing liposomes or RBC ghosts, was used to compare peroxidation and isomerization processes, allowing for a full picture of plasmalogen reactivity under free radical conditions [19]. Trans fatty acids can also be produced endogenously from free radicals and thiols, which makes them valuable biomarkers for free radical activity in the human lipidome [12]. It was recently reported that resistance to ferroptosis induced by the LP microenvironment is based on the membrane enrichment of combined SFA and TFA during remodeling [20].

### 2.3. Antioxidant Protection

The main types of antioxidants for LP are discussed in terms of structure–activity rationalization, with a focus on mechanisms, kinetics, and their potential role in modulating ferroptosis, in [17]. In this particular study, antioxidants were classified as preventive, chain-breaking, or termination-enhancing and varied from small molecules to complex enzyme systems.

A review paper in this Special Issue focuses on red blood cells (RBCs) as a biological target of oxidative reactivity due to their content of both proteins (in particular, hemoglobin) and lipids (in particular, polyunsaturated components of RBC membranes) [21]. After presenting a summary of the most relevant mechanisms and effects of oxidants in RBCs, along with a summary of the production of modified biomolecules that can act as biomarkers, the review describes an antioxidant armoire with low-molecular-weight compounds (such as ascorbate, alpha-tocopherol, and glutathione) and a large network of antioxidant proteins that can react and deactivate oxidant species (such as superoxide dismutase, glutathione peroxidase, glutathione reductase, peroxiredoxin 2, thioredoxin, thioredoxin reductase, glutaredoxins, and catalase). An excursus of the main pathological consequences of oxidative stress in RBCs is also provided. Enzymatic defense deficiencies, which cause this otherwise very resistant and flexible cell type to become fragile and degrade, are also explored [21].

Another review paper in this Special Issue focuses on diabetes mellitus, a pathological condition characterized by oxidative stress and inflammation; thus, knowledge on the molecular mechanisms of polyphenols is required to ensure their utility as nutritional antioxidants that can be used to improve the efficiency of antidiabetic treatments [22]. Diabetes mellitus includes the occurrence of this condition during pregnancy; therefore, the use of nutrition as a tool for pharmacological improvements and to optimize fetus resistance is even more justified. After a thorough description of the molecular mechanisms of reactive oxygen species generation in cells, DNA, and lipid- and protein-damaging processes, along with the individuation of biomarkers of this reactivity (such as advanced glycation-end—AGE—products), this overview of diabetes mellitus not only includes results from clinical trials but also concludes by describing pancreatic cell function, glucose homeostasis (also controlled by antioxidants), and the enzymatic antioxidant defense system. This review offers a complete evaluation of polyphenols as antioxidants, focusing on nutritional, metabolic, and clinical aspects and supporting their use in diabetes mellitus treatment [22].

Another article in this Special Issue focuses on the toxic effects of cadmium (Cd) as a potentially toxic element (PTE) deriving from pollution or industrial processes that humans can be involved with, thus causing a human health concern and the need for individuating a good antioxidant treatment [23]. In particular, liver cells are targets of Cd-induced oxidative stress, especially when this element is combined with the exposure of free fatty acids, which are known as the main compounds processed in liver cells. Moreover, melatonin (MLT), a hormonal regulator of the circadian rhythm that, as an antioxidant and cytoprotective agent, is endowed with pharmacological properties, was considered to efficiently counteract the oxidative damage. This article shows a proof of concept of the synergic activity between Cd and fatty acids for reactive oxygen species (ROS) generation in an in vitro model of liver cells (HepaRG) and intestinal epithelial cancer cells (CACO-2 cells), thus enhancing the potential for fatty acids to produce lipotoxicity and cell damage. Melatonin demonstrated its utility as a cytoprotective agent, having specific molecular mechanisms involving ERK1/2 agonism, halting cell signaling via the MAPK-ERK1/2 pathway and SAPK activation in the hepatic cell line. Cd and fatty acids induced the accumulation of cellular lipids, whereas MLT lessened the cell death induction effects both in HepaRG and CACO-2 cell lines [23]. These results should encourage further pre-clinical and clinical studies on using MLT as an antioxidant intervention option in the context of Cd exposure.

### 2.4. Oxidative and Radical Stress: Measurements and Protein Studies

Another review in this Special Issue deals with important issues related to the use of fluorescent or luminescent probes and other chemical reagents to measure oxidative and radical stress [24]. This review emphasizes the need to understand reaction pathways and, in particular, to quantify the kinetic parameters of key reactions and measure the intracellular levels and localization of probes if such reagents have to be used. Very useful information is given, including (i) a discussion on ROS in general terms, (ii) the most widely used probes for ROS, and (iii) key points to consider when using chemical probes in free radical biology.

The structural properties of some proteins (keratin, collagen, bovine and fish gelatins) exposed to gamma radiation (10 kGy) have been studied by researchers via a variety of analytical and spectroscopical techniques [25]. Protein denaturation and changes in the secondary structures have been observed. Proteins with higher degrees of ordered structures are more stable against gamma radiation and temperature. The presence of riboflavin has different effects on their secondary structures, with a stabilizing effect for keratin and fish gelatin and a destabilizing effect for bovine gelatin, observed in both irradiated and non-irradiated samples. Riboflavin facilitates the release of free radicals that further interact with the protein chains.

Despite the therapeutic and commercial success of protein therapeutics, the development of stable protein formulations can present challenges. Indeed, proteins are subjected to physical and chemical degradation. Another review in this Special Issue focuses on the primary processes of free radical formation that are relevant to pharmaceutical formulations [26]. In this review, it is reported that oxidation and the formation of free radicals represent the major pathways for the chemical degradation of pharmaceutical formulations. In particular, emphasis is placed on autoxidation, metal-catalyzed oxidation (Fenton and Fenton-like reactions), photo-degradation, and radical generation from cavitation as result of mechanical stress and various stabilizing additives like antimicrobial preservatives.

## 3. Conclusions

The articles/reviews that comprise this Special Issue and are described above focus on modified endogenous biological molecules as markers of oxidative changes or other changes initiated by free radicals, as well the structural properties of proteins formulated for storage. A broader understanding of the mechanisms and effects of oxidative stress generation in a biological context can be obtained by reading the articles of this Special Issue. The strong multidisciplinarity of this research area is well represented, allowing for readers to obtain a broad perspective of chemical, mechanistic, analytical, molecular, and structural aspects, together with an understanding of applications in biological, pharmaceutical, pre-clinical, and clinical fields. It is hoped that through exploring the articles/reviews within this collection, the readers of this Special Issue can find interesting sources of inspiration for conducting further research on free radicals and oxidative stress.
